# Roll-to-Roll Gravure-Printed SWCNT Ring Oscillator for Flexible Microfluidic Ion Sensing

**DOI:** 10.3390/nano16110660

**Published:** 2026-05-24

**Authors:** Junfeng Sun, Hyejin Park, Jinhwa Park, Sagar Shrestha, Sajjan Parajuli, Younsu Jung

**Affiliations:** 1 Research Center for Advanced Electronics Manufacturing, State Key Laboratory of Intelligent Manufacturing Equipment and Technology, School of Mechanical Science and Engineering, Huazhong University of Science and Technology, Wuhan 430074, China; sjfczyh@hust.edu.cn; 2 Research Engineering Center for R2R Printed Flexible Computer, Sungkyunkwan University, Suwon-si 16419, Republic of Korea; hyejinpark@skku.edu (H.P.); fletus05@skku.edu (J.P.); sagar@skku.edu (S.S.); sajjan@skku.edu (S.P.); 3 Department of Biophysics, Institute of Quantum Biophysics, Sungkyunkwan University, Suwon-si 16419, Republic of Korea

**Keywords:** roll-to-roll gravure, thin film transistor, ring oscillator, ion-sensing, microfluidic

## Abstract

Rapid, accurate, and scalable ion sensing technologies are highly desirable for future flexible healthcare and lab-on-a-chip applications. Here, we present a fully roll-to-roll (R2R) gravure-printed single-walled carbon nanotube complementary ring oscillator (SWCNT-cRO)-based microfluidic ion sensing platform fabricated on a flexible substrate. The proposed platform combines scalable printed complementary electronics with frequency-based ion sensing via electrostatically induced top-gating in aqueous microfluidic environments. The fabricated SWCNT-cRO devices exhibited stable oscillation characteristics, with a high device yield (>80%) and continuous manufacturing capability at a web speed of 5.4 m/min. Printable ethanolamine/zirconium acetylacetonate-based n-doping technology enabled complementary SWCNT transistor operation, while multilayer CYTOP/FG-3650 encapsulation ensured stable electrical operation under ionic aqueous conditions. After integration into a polydimethylsiloxane-based microfluidic channel, the oscillation frequency of the SWCNT-cRO was systematically modulated by Na^+^ concentration and pH. The sensing mechanism was based on electrostatically induced carrier modulation in n-type SWCNT transistors, resulting in variations in propagation delay and corresponding shifts in oscillation frequency. Compared with conventional ion-sensitive transistor platforms, the proposed approach offers scalable manufacturing, non-contact ion sensing, elimination of external reference electrodes, and direct compatibility with digital frequency-signal processing systems. This work establishes a promising strategy for future low-cost, disposable, and flexible microfluidic sensing platforms for wearable healthcare and lab-on-a-chip applications, ion sensing, and thin-film transistors.

## 1. Introduction

Ion sensing technologies capable of rapid and reliable analysis of small-volume analytes are increasingly important for applications including medical diagnostics, environmental monitoring, wearable healthcare, and lab-on-a-chip platforms. In particular, the quantitative detection of ions such as sodium (Na^+^) and hydrogen (H^+^) is essential for monitoring physiological conditions, biochemical reactions, and disease-related biomarkers [1,2,3]. Conventional potentiometric and ion-selective sensing systems have demonstrated high sensitivity and fast response characteristics; however, they often suffer from limitations including bulky architecture, limited flexibility, complex fabrication processes, and poor salability for disposable sensing systems. To overcome these limitations, various transistor-based ion sensing platforms have been extensively investigated in recent years. Silicon nanowire field-effect transistors (SiNW-FETs) have shown excellent electrical sensitivity and low detection limits owing to their high surface-to-volume ratio [4,5]. Carbon nanotube (CNT)-based ion-sensitive transistors have also attracted considerable attention because of their high carrier mobility, chemical stability, and compatibility with flexible substrates [6]. Furthermore, graphene and other two-dimensional material-based FET sensors have demonstrated superior electrical performance due to their atomically thin channel structures and high surface sensitivity [7,8]. Despite these advantages, most previously reported ion-sensitive transistor platforms rely on rigid silicon-based fabrication technologies, external reference electrodes, and sophisticated micro/nanofabrication processes, which significantly limit their applicability to large-area, low-cost, and mechanically flexible sensing systems.

Recently, printed electronics based on roll-to-roll (R2R) manufacturing processes have emerged as promising alternatives for realizing scalable and cost-effective flexible electronic systems. R2R gravure printing offers several advantages including high throughput, continuous manufacturing capability, low material consumption, and compatibility with flexible substrates [9,10]. Owing to these advantages, printed thin-film transistors (TFTs), sensors, logic circuits and wearable electronic devices have been actively explored for next-generation disposable electronic applications [11,12,13,14,15]. Nevertheless, the realization of fully printed ion sensing platforms based on integrated printed circuits remains challenging because stable operation under aqueous environments, device uniformity, and scalable complementary circuit fabrication are difficult to achieve simultaneously. Among various printed electronic architectures, ring oscillators have attracted considerable interest because their oscillation frequency is highly sensitive to changes in transistor electrical characteristics, enabling direct signal transduction through frequency modulation [16,17,18,19]. Compared with conventional current-based sensing methods, frequency-based sensing systems provide simplified signal processing and enhanced compatibility with digital electronics. However, fully printed complementary ring oscillator-based ion sensing systems integrated with microfluidic platforms have rarely been reported, particularly using scalable R2R manufacturing approaches.

In this study, we present a scalable microfluidic ion sensing platform based on a fully R2R-printed single-walled carbon nanotube complementary ring oscillator (SWCNT-cRO). The proposed SWCNT-cRO was fabricated entirely through a continuous R2R gravure printing process on a flexible polyethylene terephthalate (PET) substate. Ethanolamine (EA) and zirconium acetylacetonate (ZAcAc)-based n-doping ink was utilized to realize complementary SWCNT-TFT circuits through a fully printing process. To ensure stable operation under ionic aqueous environments, multilayer encapsulation based on CYTOP and FG-3650 was introduced. Furthermore, a polydimethylsiloxane (PDMS)-based micro fluidic channel was integrated onto the printed SWCNT-cRO to realize real-time ion monitoring under fluidic conditions. The proposed sensing platform demonstrated stable frequency modulation behavior depending on Na^+^ concentration and pH variation, thereby establishing a promising strategy for future low-cost disposable, and flexible biosensing systems based on scalable printed electronics.

Compared with previously reported printed ion sensors, the proposed platform offers several unique advantages, including scalable manufacturing, oscillator-based signal transduction, non-contact electrostatic sensing, and direct compatibility with digital frequency readout systems. Furthermore, the demonstrated device reproducibility and statistical yield over continuously printed PET webs highlight the feasibility of high-throughput manufacturing for disposable sensing electronics.

## 2. Materials and Methods

### 2.1. Materials

Silver nanoparticle-based conductive ink, BaTiO_3_-based dielectric ink, and SWCNT-based p-type semiconducting ink (SWCNT diameter: 1–1.2 nm; length: ~20 μm) were formulated according to our previous report [15]. CYTOP (SP2 type, Asahi Glass Co., Tokyo, Japan) and FG-3650 TH-80 (FluoroTechnology, Aichi, Japan) were used as encapsulation materials. EA (ACS reagent, 99.0%), zirconium acetylacetonate (97%, MW = 487.66 g/mol), and TiO_2_ nanoparticles (<25 nm) were purchased from Sigma-Aldrich (MO, USA) and used without further purification. Diethylene glycol monobutyl ether (99.5%) was purchased from Daejung Chemical & Materials (Gyeonggi-do, Republic of Korea).

For the formulation of the n-doping ink, 1 g of zirconium acetylacetonate was first dissolved in 20 mL of diethylene glycol monobutyl ether using a bath sonicator for 8 h. Subsequently, 5 mL of EA was added to the solution, followed by additional sonication for 30 min. Finally, 0.2 g of TiO_2_ nanoparticles was dispersed into the prepared solution using a bath sonication process for 30 min to obtain the final n-doping ink formulation.

### 2.2. Methods

A customized R2R gravure printing system with two printing units (i-Pen Co., Gyeonggi-do, Republic of Korea) was employed in this study. The fabrication process of the SWCNT-cRO was conducted similarly to our previous report. Briefly, the gate electrodes for the p- and n-type SWCNT thin-film transistors (TFTs) were first printed onto a PET substrate (100 μm thickness, 250 mm width; AH71D (SKC, Gyeonggi-do, Republic of Korea). Subsequently, the dielectric layer was continuously printed in the second printing unit. The SWCNT active layer was then overprinted on the gate/dielectric structure, followed by printing of the source and drain electrodes on top of the SWCNT layer. Finally, the formulated n-doping ink was selectively overprinted onto the n-channel region of the SWCNT-TFTs.

All electronic inks were printed at a printing speed of 5.4 m/min with a nip force of 60 N. After completion of the printing process, the fabricated SWCNT-cRO devices were encapsulated with CYTOP and FG-3650 TH-80 using a spin-coating process (ACE-200, DONG AH TRADE Corp., Seoul, Republic of Korea).

For fabrication of the microfluidic channel, a reusable positive mold was machined from Teflon owing to its excellent anti-adhesion property. The designed channel consisted of a straight geometry with dimensions of 2 mm in width, 1 mm in height, and 30 mm in length. The PDMS precursor and curing agent (Sylard 184, Dow Corning, MI, USA) were mixed at a weight ratio of 10:1 and degassed under vacuum for 30 min to remove trapped air bubbles. The prepared PDMS mixture was subsequently poured into the Teflon mold and thermally cured at 80 °C for 2 h. After curing, the PDMS microfluidic channel was carefully peeled off from the mold without structural damage for channel collapse. The fabricated microchannel was then laminated onto the encapsulated SWCNT-cRO device using adhesive bonding. The relatively large millimeter-scale straight channel structure enabled stable ionic liquid transport while minimizing fabrication complexity and demolding issues. PDMS was selected as the channel material because of its flexibility and suitability for sensing application.

All measures were carried out in ambient conditions. The output signals of devices were acquired using a digital phosphor oscilloscope (DPD 4034, Tektronix, OR, USA) with a DC power supply (AK-3003D, ICAN Co., Ltd., Daejeon, Republic of Korea). Furthermore, the transfer and output characteristics of SWCNT-cRO were measured using a KEITHLEY 4200 semiconductor characterization system (Tektronix, OR, USA) with an ambient probe station (MST-4000A, MS TECH Co., Ltd., Incheon, Republic of Korea). A Syringe Pump (Longer Pump Co., Hebei, China) was used to inject the analytes.

## 3. Results and Discussion

### 3.1. Preparation and Characterization of R2R Gravure-Printed SWCNT-cRO

**Figure 1:**
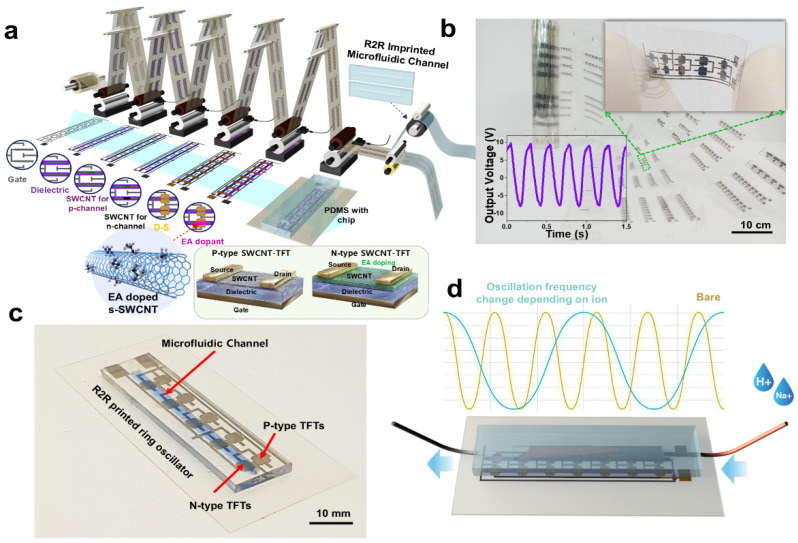
(**a**) Schematic illustration of the R2R gravure printing process and device architecture of the SWCNT-cRO integrated with a microfluidic platform. (**b**) Optical image of the fully R2R gravure-printed SWCNT-cRO arrays with different stage numbers (inset: representative oscillation output signal of the printed 5-stage SWCNT-cRO). (**c**) Optical image of the SWCNT-cRO laminated with a PDMS-based microfluidic chip. (**d**) Schematic illustration of the ion sensing mechanism of the SWCNT-cRO integrated with a PDMS-based microfluidic channel.

In general, the R2R gravure printing process was similar to that reported in our previous work [15]. A bottom-gate approach was adopted to fabricate the SWCNT-cRO on a PET roll, as shown in Figure 1a. A practical printing speed of 5.4 m/min was maintained to print all six layers, including the gate, dielectric, SWCNT active, drain source, and n-doping layers (Figure 1a inset image), followed by CYTOP/FG-3650 encapsulation via a spin coating process. Furthermore, SWCNT-cRO circuits with four different stage configurations (3, 5, 7, and 9 stages) were designed and fabricated, as shown in Figure S1, to optimize the output frequency and voltage characteristics. The dimensional parameters of the designed SWCNT-cRO, including gate width, channel length, and channel width, are summarized in Table 1. A representative oscillation output signal from the printed 5-stage SWCNT-cRO is presented in the inset of Figure 1b. Moreover, Figure 1c shows an optical image of the integrated system consisting of the SWCNT-cRO and a PDMS-based microfluidic chip [20,21,22,23]. The SWCNT-cRO was composed of complementary p- and n-type SWCNT-TFTs, enabling low-power consumption and stable oscillation behavior. The operating principle of ion sensing based on frequency modulation is illustrated in Figure 1d. The presence of ions (e.g., H^+^, Na^+^) within the microfluidic channel alters the electrical characteristic of the n-type SWCNT channel, leading to a shift in oscillation frequency. The frequency variation enables label-free and real-time ion detection.

**Figure 2:**
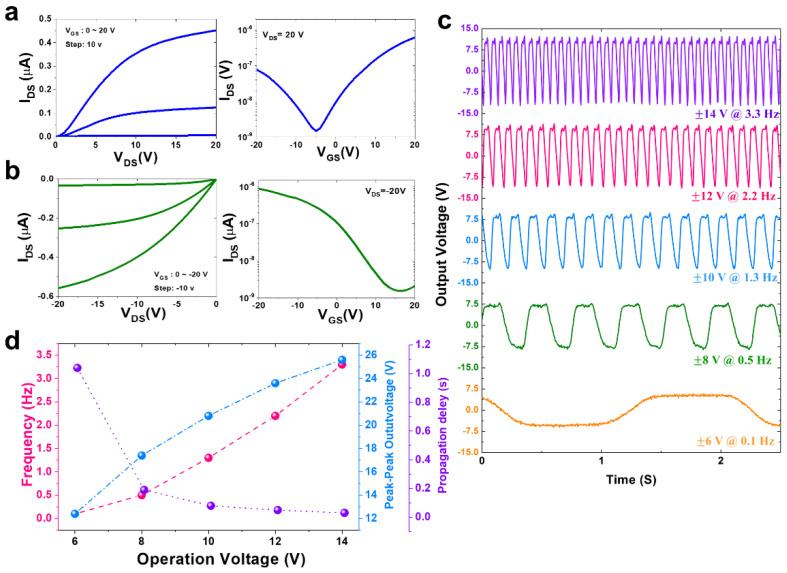
(**a**) Representative output and transfer characteristics of the printed n-type SWCNT-TFT. (**b**) Representative output and transfer characteristics of the printed p-type SWCNT-TFT. (**c**) Oscillation output signals of the printed 5-stage SWCNT-cRO measured at V_DD_ of ±6, ±8, ±10, ±12 and ±14 V. (**d**) Dependence of oscillation frequency, peak-to-peak output voltage (V_PP_), and propagation delay on the applied V_DD_.

Since the concentration of SWCNTs in the formulated ink significantly influences the electrical performance of the TFT, the effect of SWCNT concentration in the semiconducting ink was further investigated. A higher yield was achieved using an SWCNT concentration of 0.33 mg/mL for n-type SWCNT-TFTs and 0.27 mg/mL for p-type SWCNT-TFTs. The semiconducting characteristics of the SWCNT were confirmed by ultraviolet-visible-near-infrared (UV-Vis-NIR) spectroscopy, as shown in Figure S2a, while the formation of an SWCNT random network was observed in the active channels of the printed SWCNT-TFTs (Figure S2b). The printed p-type SWCNT-TFTs exhibited an average ON/OFF ratio of 10^2.8^, a hole mobility of 0.1 cm^2^/Vs, and threshold voltage (Vth) of +8 V. In contrast, the printed n-type SWCNT-TFTs showed an average ON/OFF ratio of 10^2.6^, an electron mobility of 0.08 cm^2^/Vs, and a Vth of −3 V. Representative electrical characteristics of the printed p- and n-type TFTs are shown in Figure 2a and Figure 2b, respectively.

The realization of a fully printed SWCNT-cRO on a flexible substrate was achieved through n-doping of the intrinsically p-type SWCNT channel, as illustrated in Figure 2a. In general, exposure to oxygen and water molecules causes SWCNTs with ambipolar characteristics to exhibit p-type behavior [24,25]. However, the adsorption of amine-rich molecules, such as EA, provides sufficient electron donation, resulting in hole depletion within the semiconducting p-type TFT channel. To convert this p-type behavior to n-type, EA- and ZAcAc-based doping inks were formulated (see Section 2). The incorporation of ZAcAc improves the dewetting characteristics on the printed SWCNT channel surface. In addition, diethylene glycol monobutyl ether was employed to optimize the rheological properties of the ink, including viscosity and surface tension. The formulated n-doping ink exhibited a viscosity of 12 mPas and a surface tension of 32 mN/m.

Furthermore, to enhance the printability of the EA-ZAcAc-based n-doping ink, TiO_2_ nanoparticles were incorporated as an inorganic additive using a simple bath sonication process. To verify the doping effect of the formulated n-type ink, roll-to-plate (R2P) printing was first employed to evaluate the conversion behavior of the p-type channel after drying at 80 °C for 5 min. A clear conversion from p-type to n-type characteristics was observed, as shown in Figure S3. The optimized n-doping ink was subsequently applied to the R2R gravure printing process without further modification and was successfully deposited on top of the SWCNT layer, forming an approximately 80 nm-thick doping layers (Figure S4). The electrical characteristics of the SWCNT-TFTs confirmed effective doping by the optimized n-type ink, demonstrating successful polarity conversion of the intrinsically p-type TFTs into n-type devices, as shown in Figure 2a,b.

To evaluate manufacturing uniformity, 25 SWCNT-cRO devices were randomly selected from different positions along the 10 m long printed PET roll. The device yield was defined as the percentage of devices exhibiting stable oscillation with a measurable peak-to-peak output voltage (V_PP_) at V_DD_ = ±10 V. The results are summarized in Table 1, which includes statistical analyses of the oscillatory yield for SWCNT-cRO with different stage numbers and SWCNT-TFT dimensions. Among the tested configurations, the 5-stage SWCNT-cRO fabricated using 0.33 mg/mL SWCNT ink for n-channel TFTs and 0.27 mg/mL SWCNT ink for p-channel TFTs exhibited the highest oscillation yield of 82%. The optimized device geometry consisted of a gate width of 400 μm, a channel length of 150 μm, and a channel width of 1600 μm for the p-type TFTs, while the n-type TFTs had a channel length of 100 μm and a channel width of 1600 μm. In the SWCNT-cRO inverter chain, the oscillation frequency exhibited excellent linearity with the applied supply voltage (V_DD_), as shown in Figure 2c,d.

**Figure 3:**
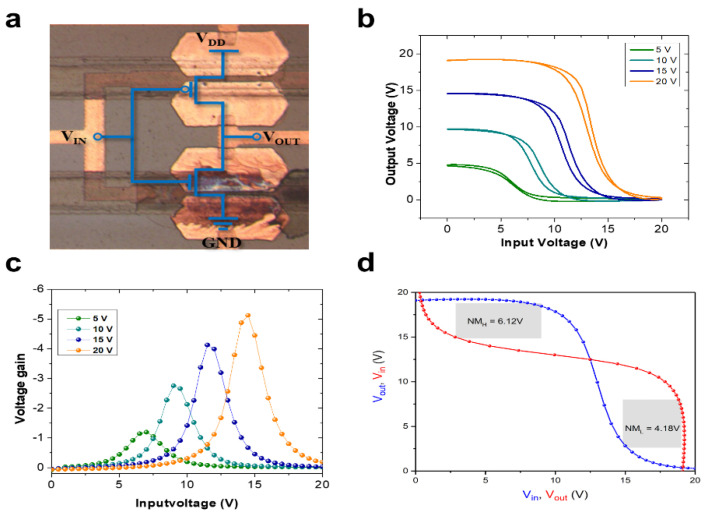
(**a**) Optical image and circuit schematic of the bottom-gate inverter composed of complementary p- and n-type SWCNT-TFTs. (**b**) Voltage transfer characteristics (VTCs) of the inverter measured at V_DD_ of 5, 10, 15, and 20 V. (**c**) Voltage gain characteristics of the inverter under different V_DD_ conditions (5, 10, 15, and 20 V). (**d**) Noise margin analysis of the fully printed inverter at V_DD_ = 20 V.

The electrical characteristics of the inverter, which serve as the fundamental building block of the SWCNT-cRO, were further analyzed in terms of voltage transfer characteristics (VTC), voltage gain, and noise margin [26,27,28,29]. Figure 3a presents the optical image and circuit schematic of the inverter circuit fabricated through the R2R printing process. The VTCs of the proposed inverter were measured under ambient conditions at four supply voltages V_DD_ of 5, 10, 15, and 20 V, as shown in Figure 3b. The measured results demonstrate typical inverter behavior with a full rail-to-rail output voltage swing. Specifically, the output voltages (V_out_) reached 4.8, 9.7, 14.6, and 19.2 V at V_DD_ values of 5, 10, 15, and 20 V, respectively. The maximum trip point was observed at V_DD_ = 20 V and was approximately 1.25 times higher than the ideal trip-point value of 10 V. Under the same operating condition, the fully R2R-printed inverter exhibited a maximum voltage gain $\left(\Delta V_{out}/\Delta V_{in}\right)$ of 5.12, as shown in Figure 3c. In addition, the maximum noise margin (~6.12 V) obtained at V_DD_ = 20 V corresponded to 61.2% of V_DD_/2 (Figure 3d).

The propagation delay and oscillation frequency of the ring oscillator were systematically investigated as functions of the input bias voltage. Stable inverter operation with clear oscillation signals was observed (Figure 2c), while the propagation delay decreased from approximately 1 s to 30 ms with increasing V_DD_. The propagation delay (t_p_) was determined according to the following:

$$t_{p}=\frac{1}{2fN}$$

where t_p_ is the propagation delay, f is the oscillation frequency, and N is the number of stages (N = 5). As expected from this relationship, both the oscillation frequency and output amplitude increased linearly as the input bias voltage increased from ±6 V to ±14 V (Figure 2c,d). At an input bias voltage of ±6 V, the SWCNT-cRO exhibited a low oscillation frequency of 0.1 Hz with a peak-to-peak voltage (V_pp_) of 12.4 V. As the bias voltage increased, the oscillation frequency increased up to 3.3 Hz, accompanied by a $V_{pp}$ of 25.6 V. The observed linear relationship between the input bias voltage and oscillation frequency demonstrates the feasibility of directly employing the fully R2R-printed ring oscillator as a flexible ion sensing platform.

### 3.2. Ion Monitoring Test for R2R Gravure-Printed SWCNT-cRO

**Figure 4:**
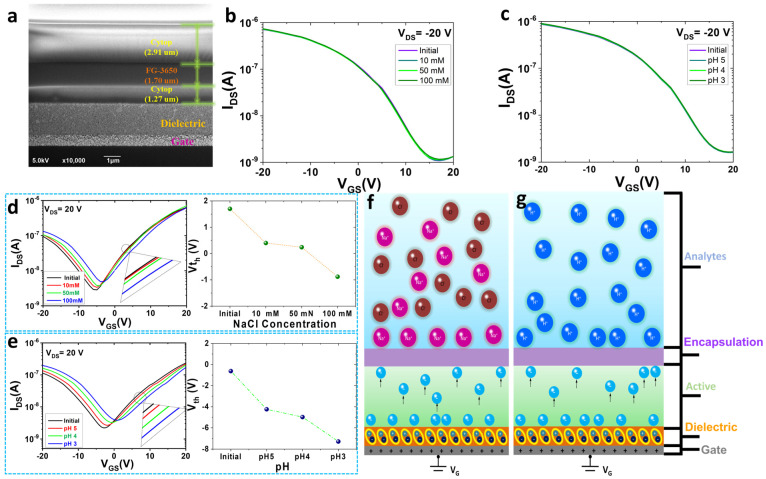
(**a**) Focused ion beam-scanning electron microscopy (FIB-SEM) cross-section of the optimized CYTOP/FG-3650 multilayer encapsulation on the SWCNT-TFTs. (**b**) Transfer characteristics of encapsulated p-type SWCNT-TFTs under NaCl aqueous solutions ranging from 10 to 100 mM. (**c**) Transfer characteristics of encapsulated p-type SWCNT-TFTs under pH buffer solutions ranging from pH 3 to pH 5. (**d**) Transfer characteristics of doped n-type SWCNT-TFTs under different NaCl concentrations and the corresponding Vth variation. (**e**) Transfer characteristics of doped n-type SWCNT-TFTs under different pH buffer solutions and the corresponding Vth variation. (**f**) Schematic illustration of the ion sensing mechanism of the n-type SWCNT-TFT for NaCl analytes. (**g**) Schematic illustration of the ion sensing mechanism of the n-type SWCNT-TFT for pH analytes.

A multilayer encapsulation strategy was investigated to stabilize the output signal and protect the device from analyte penetration. Initially, two layers of CYTOP and two layers of FG-3650 were deposited via spin coating, and their stability was evaluated by applying deionized (DI) water as a precursor analyte to both p- and n-type channels [27,30,31,32]. As shown in Figure S5a, the Vth of the p-type SWCNT-TFTs remained unchanged, whereas the n-type SWCNT-TFTs exhibited a positive shift in Vth upon DI water exposure. To achieve stable electrical operation under DI water loading, additional CYTOP layers were subsequently introduced, as shown in Figure S5b–d. Through this optimization process, a compounded multilayer encapsulation structure consisting of CYTOP and FG-3650 with a total thickness of 5.88 μm was achieved (Figure 4a), providing excellent stability against DI water exposure.

To evaluate ion sensitivity, an aqueous NaCl solution (50 mM) and a pH buffer solution (pH 4) were applied onto the SWCNT-TFTs, and the resulting $V_{th}$ shifts were monitored (Figure S5e,f). Furthermore, to systematically investigate the sensing performance of the proposed ion sensing platform, NaCl solutions with concentrations ranging from 10 to 100 mM and pH buffer solutions ranging from pH 3 to pH 5 were employed as analytes. As expected, the encapsulated p-type SWCNT-TFTs exhibited highly stable electrical characteristics under both NaCl and pH analytes, regardless of NaCl concentration or pH value (Figure 4b,c). In contrast, the doped n-type SWCNT-TFTs showed clear linear sensitivity, characterized by a positive shift in Vth and slight current degradation as the NaCl concentration increased and the pH value decreased (Figure 4d,e). Specifically, the $V_{th}$ shifted from 1.7 V to −0.9 V for the NaCl analyte and from 0.6 V to −7.2 V for the pH analyte.

Regarding the ion sensing mechanism, the n-type electrical behavior of the SWCNT-TFTs originated from the electron-donating effect of the amine-based doping molecules, including EA, which suppress hole transport in the intrinsically p-type SWCNT network [33,34,35,36,37]. As a result, the doped SWCNT channel exhibits n-channel transport characteristics under ambient conditions. When ionic analytes such as Na^+^ and H^+^ are introduced into the PDMS microfluidic channel, the ions accumulate near the surface of the multilayer encapsulation consisting of CYTOP and FG-3650, as schematically illustrated in Figure 4f,g. Similar to the electrostatic sensing mechanism reported in CYTOP-coated SWCNT-TFT sensing systems [33], the accumulated ionic charges induce an additional electrostatic potential across the encapsulation layer, which acts as top-gating electric field on the underlying SWCNT channel.

**Figure 5:**
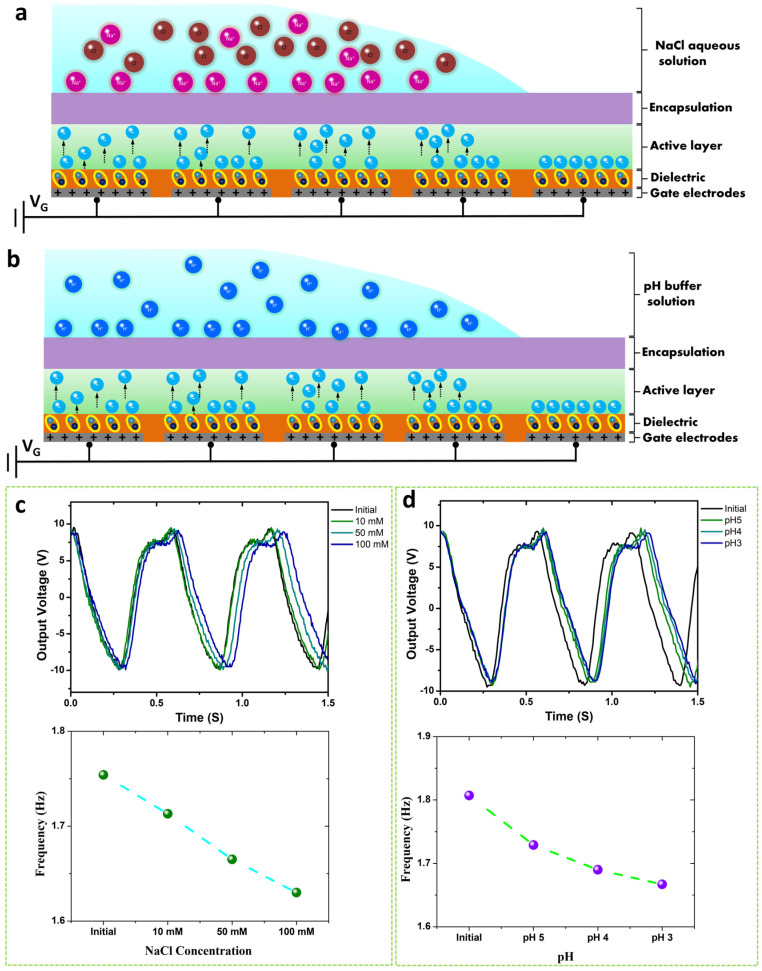
(**a**) Schematic illustration of the sensing mechanism of the microfluidic SWCNT-cRO platform under a NaCl aqueous solution flow through the n-type SWCNT-TFT region. (**b**) Schematic illustration of the sensing mechanism of the microfluidic SWCNT-cRO platform under pH buffer solution flow through the n-type SWCNT-TFT region. (**c**) Oscillation output signals and corresponding frequency variation of the printed SWCNT-cRO under different NaCl concentrations (10–100 mM). (**d**) Oscillation output signals and corresponding frequency variation of the printed SWCNT-cRO under different pH buffer solutions (pH 3–5).

This electrostatic top-gating effect modulates the carrier concentration of the amine-doped SWCNT channel through capacitive coupling without direct contact between aqueous analytes and the active semiconducting layer. As the concentration of cationic analytes increases, the induced positive electrostatic potential weakens the electron-doping effect of the amine molecules and gradually restores hole transport characteristics in the SWCNT network. Consequently, the electrical characteristics of the doped SWCNT-TFT shift toward intrinsic p-type behavior, resulting in a positive Vth shift and degradation of the n-channel current (Figure 5a,b). The modulation of the transistor current directly affects propagation delay of the SWCNT-cRO stages, since the propagation delay of the ring oscillator is inversely proportional to the driving current of the TFTs [38].

$$t_{d}=\frac{C_{total}V_{DD}}{I_{D}}$$

where t_d_ is the stage propagation delay, C_total_ is the parasitic capacitance, V_DD_ is the supply voltage, and I_D_ is the drain current. The reduction on n-channel current under higher ion concentrations increases the propagation delay of each inverter stage. Consequently, the oscillation frequency of the SWCNT-cRO decreases systematically with an increasing NaCl concentration and decreasing pH values, as observed in Figure 5c,d. This sensing mechanism differs from conventional ISFET-based approaches because the proposed platform does not require direct exposure of the semiconducting channel to the electrolyte or the use of an external reference electrode. Instead, the ionic analytes modulate the oscillation characteristics of the SWCNT-cRO through an electrostatically induced top-gating effect across the encapsulation layer, enabling stable and scalable non-contact ion sensing under aqueous microfluidic environments.

Although the present work primarily focuses on Na^+^- and H^+^-dependent frequency modulation, the sensing mechanism is fundamentally governed by electrostatically induced carrier modulation arising from ionic charge accumulation near the encapsulation surface. Therefore, other cationic species such as K^+^, Ca^2+^, and Mg^2+^ are also expected to influence the oscillation characteristics through similar electrostatic interactions. However, due to differences in ionic valence, hydration radius, mobility, and interfacial adsorption behavior, distinct frequency responses may occur depending on ion species and concentration. Detailed selectivity and cross-sensitivity analyses toward various ionic species will be investigated systematically in future studies.

## 4. Conclusions

In this study, we demonstrated a scalable microfluidic ion sensing platform based on a fully R2R gravure-printed SWCNT-cRO fabricated on a flexible PET substrate. Furthermore, multilayer encapsulation based on CYTOP and FG-3650 enabled reliable operation under aqueous microfluidic environments. The proposed sensing platform utilized electrostatically induced top-gating effects generated by ionic analytes flowing through the PDMS microfluidic channel. The accumulated ionic charges on the encapsulation surface modulated the carrier transport characteristics of the n-type SWCNT-TFTs, resulting in systematic variations in propagation delay and oscillation frequency. Consequently, the SWCNT-cRO exhibited clear frequency responses depending on NaCl concentration and pH variation, demonstrating the feasibility of frequency-based ion sensing using fully printed complementary circuits.

Compared with conventional ion-sensitive transistor platforms, the proposed approach offers several important advantages, including scalable R2R manufacturability, flexible device integration, non-contact sensing operation, and elimination of external reference electrodes. In particular, the use of oscillation frequency as the sensing signal provides direct compatibility with digital signal processing systems and wireless sensing architectures, which is advantageous for low-power and portable sensing applications. In addition, the statistical uniformity and stable operation of the fully printed SWCNT-cROs over the continuously printed PET web highlight the potential of the proposed platform for high-throughput and low-cost manufacturing of disposable sensing electronics. The demonstrated device architecture can be further extended to multiplexed sensing systems, wearable healthcare platforms, environmental monitoring systems, and IoT-based smart sensing applications.

Overall, this work established a practical strategy for integrating scalable printed electronics, complementary SWCNT logic circuits, and microfluidic sensing technologies into a unified flexible sensing platform. The proposed SWCNT-cRO-based sensing architecture provides a promising pathway toward next-generation printable and large-area intelligent sensor systems.

## Figures and Tables

**Table 1:** Summary of the oscillation yield and TFT dimensions of the R2R gravure-printed SWCNT-cROs with different stage configurations and SWCNT concentrations for p- and n-channel TFT fabrication. The table summarizes the gate width, channel length (CL), channel width (CW), and corresponding device yield of the 3-, 5-, 7-, and 9-stage SWCNT-cROs, based on a statistical analysis of 25 devices for each fabrication condition.

Ink Information for N-Type and SWCNT-TFT Dimensions			Oscillating Yield			
SWCNT Concentration of n-Channel	SWCNT Concentration of n-Channel	SWCNT-TFT Dimensions	3 Stages	5 Stages	7 Stages	9 Stages
0.27 mg/mL	0.27 mg/mL	Gate width: 400 µm CL: p-type 150 µm, n-type 100 µm CW: p-type 1600 µm, n-type 1600 µm	35%	1%	44%	13%
		Gate width: 400 µm CL: p-type 150 µm, n-type 100 µm CW: p-type 1600 µm, n-type 2000 µm	50%	45%	38%	38%
		Gate width: 400 µm CL: p-type 150 µm, n-type 100 µm CW: p-type 1600 µm, n-type 3000 µm	44%	27%	38%	19%
		Gate width: 400 µm CL: p-type 150 µm, n-type 80 µm CW: p-type 1600 µm, n-type 3000 µm	25%	1%	13%	0%
	0.33 mg/mL	Gate width: 400 µm CL: p-type 150 µm, n-type 100 µm CW: p-type 1600 µm, n-type 1600 µm	55%	82%	50%	64%
		Gate width: 400 µm CL: p-type 150 µm, n-type 100 µm CW: p-type 1600 µm, n-type 2000 µm	55%	55%	64%	71%
		Gate width: 400 µm CL: p-type 150 µm, n-type 100 µm CW: p-type 1600 µm, n-type 3000 µm	50%	36%	78%	78%
		Gate width: 400 µm CL: p-type 150 µm, n-type 80 µm CW: p-type 1600 µm, n-type 3000 µm	45%	45%	78%	78%

## Data Availability

Data is contained within the article or Supplementary Materials. Further inquiries can be directed to the corresponding author.

## Supplementary Materials

The following supporting information can be downloaded at: https://www.mdpi.com/article/10.3390/nano16110660/s1, Figure S1: Layout design of the SWCNT-cROs with different stage configurations (3-, 5-, 7-, and 9-stage ring oscillators) and TFT dimensions for R2R gravure cylinder fabrication. The inset shows the unit inverter structure composed of complementary p- and n-type SWCNT-TFTs; Figure S2: (a) UV–Vis–NIR absorption spectrum of the semiconducting SWCNT ink with a concentration of 0.27 mg/mL. (b) SEM image of the randomly distributed SWCNT network formed in the printed TFT channel region; Figure S3: (a) Output and transfer characteristics of the pristine p-type SWCNT-TFT fabricated by the R2R gravure printing process. (b) Output and transfer characteristics of the n-type SWCNT-TFT after EA-based n-doping realized through the R2P printing process; Figure S4: Focused ion beam–scanning electron microscopy (FIB–SEM) cross-sectional image of the printed n-doped SWCNT-TFT channel, showing the formation of the printed n-doping layer on top of the SWCNT active layer; Figure S5: (a–d) Transfer characteristics of the R2R gravure-printed p- and n-type SWCNT-TFTs after DI water loading under different spin-coated encapsulation conditions consisting of 4, 5, 6, and 7 CYTOP layers with 2 FG-3650 layers. (e) Transfer characteristics of the R2R gravure-printed p- and n-type SWCNT-TFTs after exposure to DI water and 50 mM NaCl solution under the optimized encapsulation condition consisting of 7 CYTOP layers and 2 FG-3650 layers. (f) Transfer characteristics of the R2R gravure-printed p- and n-type SWCNT-TFTs after exposure to DI water and pH 4 buffer solution under the optimized encapsulation condition consisting of 7 CYTOP layers and 2 FG-3650 layers.
