# Supplementary material for: Roll-to-Roll Gravure-Printed SWCNT Ring Oscillator for Flexible Microfluidic Ion Sensing
Source: Nanomaterials (Basel). 2026 May 24;16(11):660. doi: 10.3390/nano16110660 (PMC13258646; doi:10.3390/nano16110660)
Supplement: Supplementary file 1 [file nanomaterials-16-00660-s001.zip › nanomaterials-4293599-supplementary.pdf]

Supporting Information

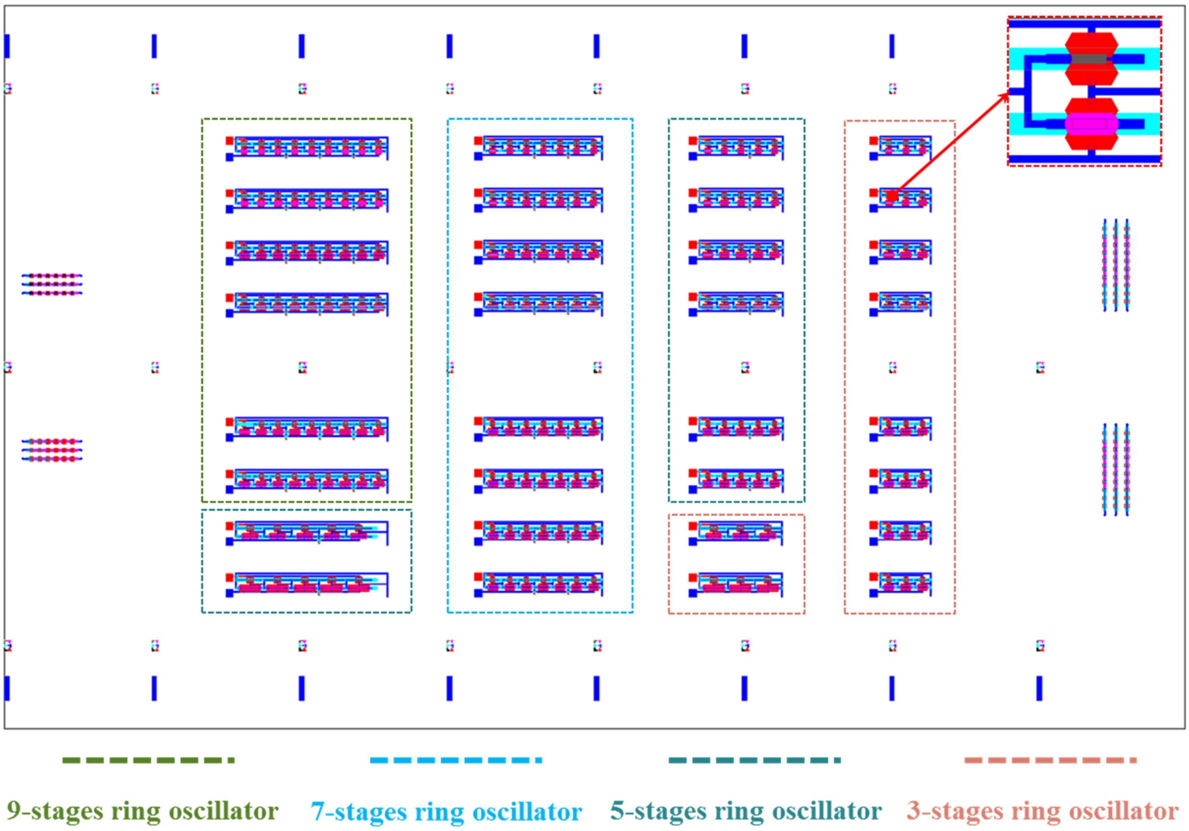

Figure S1. Layout design of the SWCNT-cROs with different stage configurations (3-, 5-, 7-, and 9-stage ring oscillators) and TFT dimensions for R2R gravure cylinder fabrication. The inset shows the unit inverter structure composed of complementary p- and n-type SWCNT-TFTs.

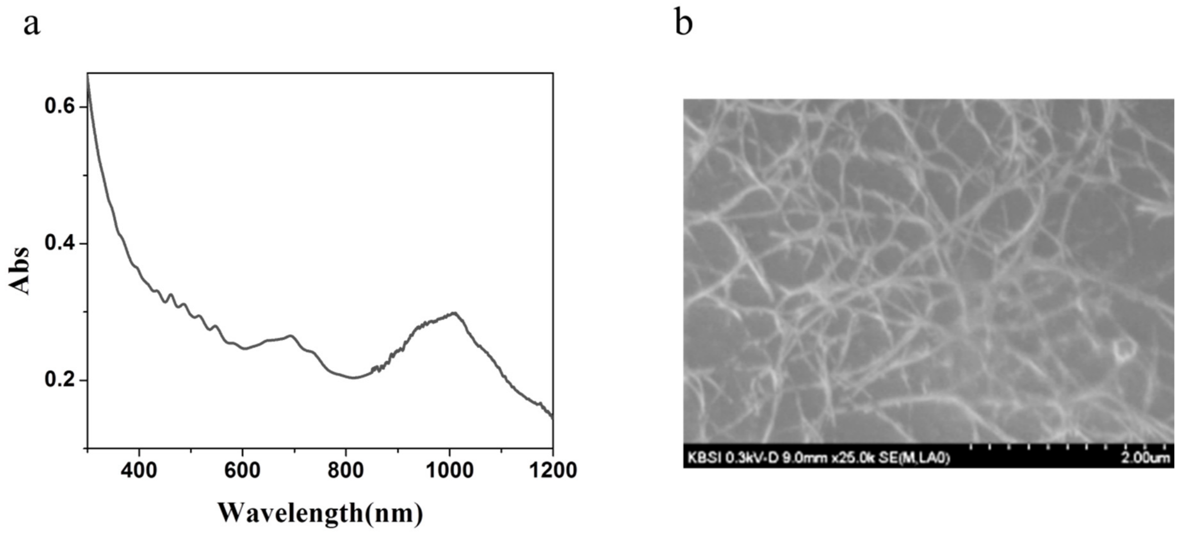

Figure S2. a) UV–Vis–NIR absorption spectrum of the semiconducting SWCNT ink with a concentration of 0.27 mg/mL. b) SEM image of the randomly distributed SWCNT network formed in the printed TFT channel region..

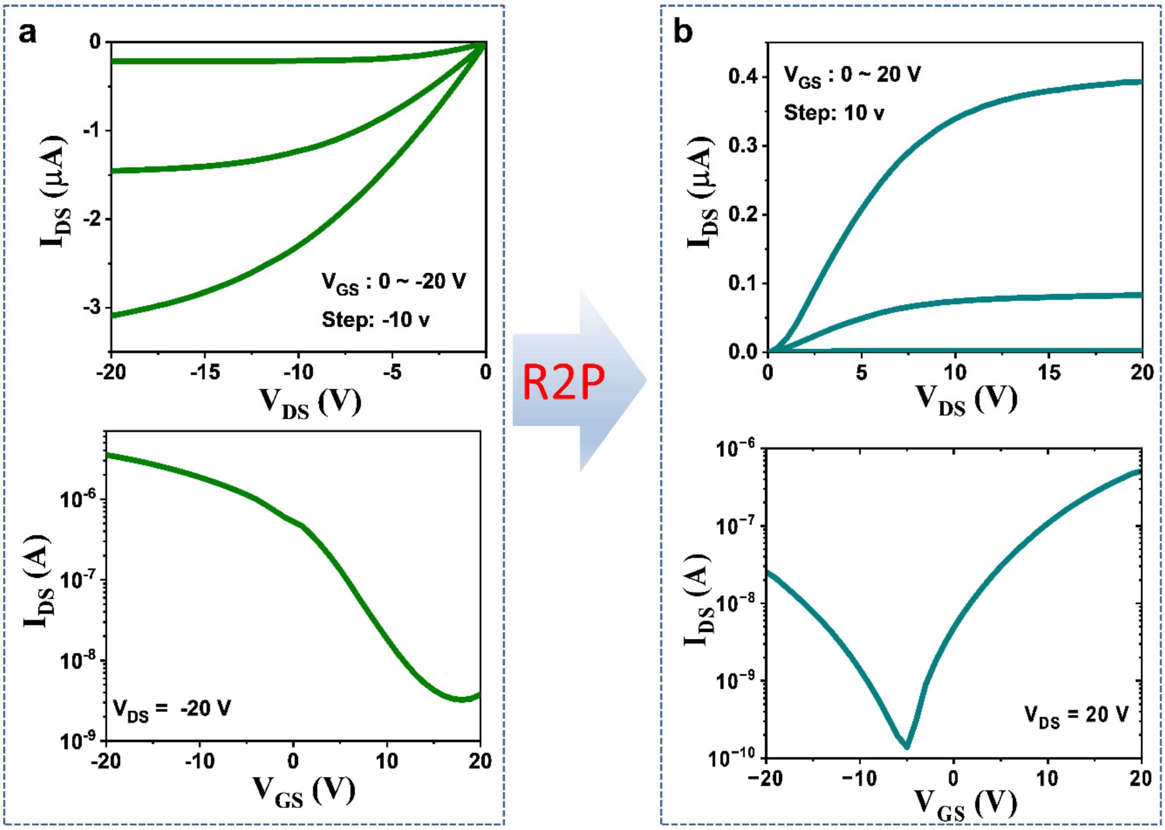

Figure S3. a) Output and transfer characteristics of the pristine p-type SWCNT-TFT fabricated by the R2R gravure printing process. b) Output and transfer characteristics of the n-type SWCNT-TFT after EA-based n-doping realized through the R2P printing process.

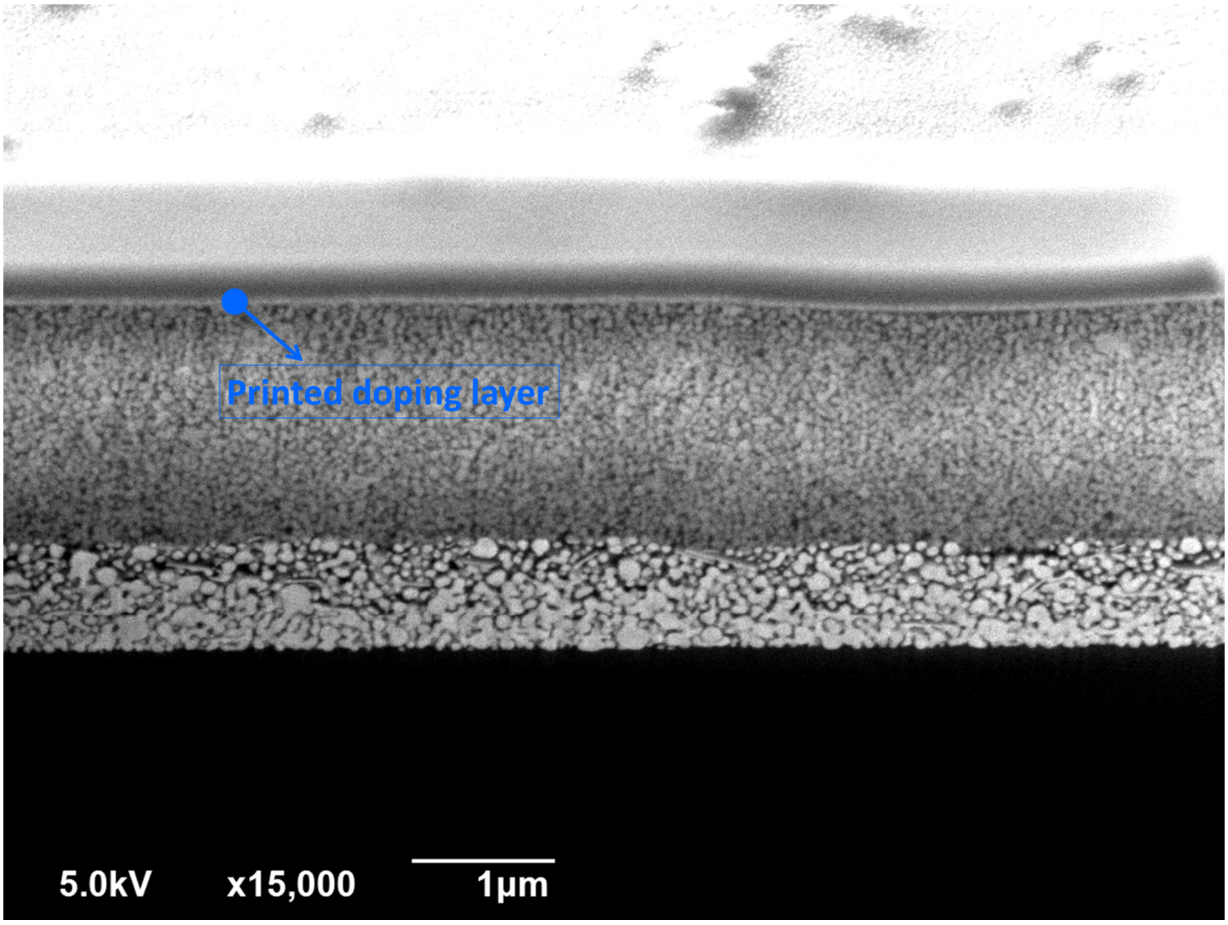

Figure S4. Focused ion beam–scanning electron microscopy (FIB–SEM) cross-sectional image of the printed n-doped SWCNT-TFT channel, showing the formation of the printed n-doping layer on top of the SWCNT active layer.

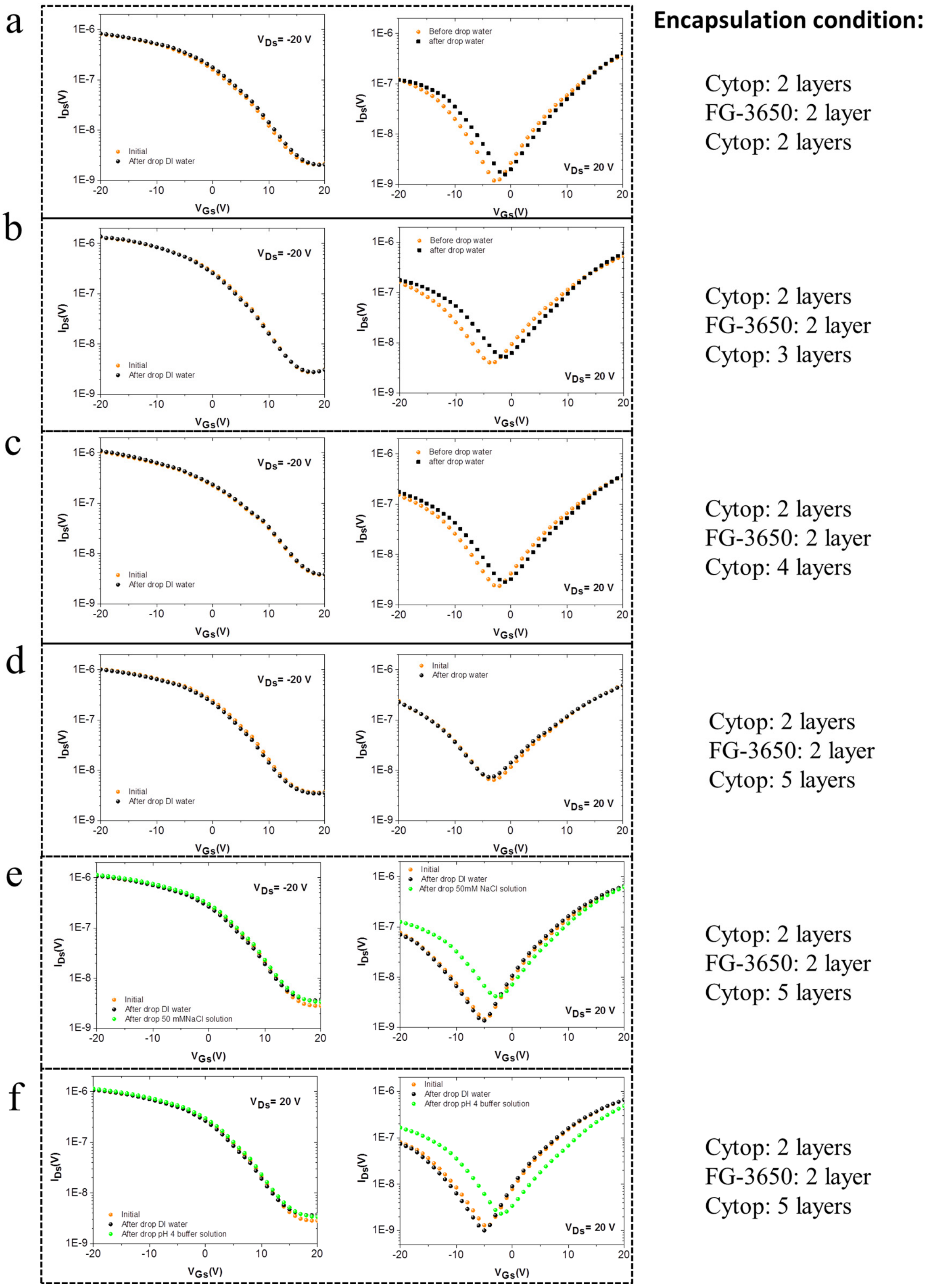

Figure S5. a–d) Transfer characteristics of the R2R gravure-printed p- and n-type SWCNT-TFTs after DI water loading under different spin-coated encapsulation conditions consisting of 4, 5, 6, and 7 CYTOP layers with 2 FG-3650 layers. e) Transfer characteristics of the R2R gravure-printed p- and n-type SWCNT-TFTs after exposure to DI water and 50 mM NaCl solution under the optimized encapsulation condition consisting of 7 CYTOP layers and 2 FG-3650 layers. f) Transfer characteristics of the R2R gravure-printed p- and n-type SWCNT-TFTs after exposure to DI water and pH 4 buffer solution under the optimized encapsulation condition consisting of 7 CYTOP layers and 2 FG-3650 layers.
